# Single-Stage Full-Thickness Scalp Reconstruction Using Acellular Dermal Matrix and Skin Graft

**Published:** 2011-01-25

**Authors:** Yoon S. Chun, Kapil Verma

**Affiliations:** ^a^Division of Plastic and Reconstructive Surgery, Department of Surgery, Brigham and Women's Hospital/Faulkner Hospital, Boston, MA; ^b^Harvard Medical School, Boston, MA

## Abstract

Full-thickness scalp defects with exposed calvarium pose a reconstructive challenge, particularly in patients with extensive comorbidities. A single-stage acellular dermal matrix and split-thickness skin graft reconstruction represents a simple and valuable surgical approach to achieving durable scalp coverage without requiring a donor-site or prolonged treatment. Reconstruction of full-thickness scalp defects with exposed calvarium is a difficult problem that generally requires local or free flap reconstruction. However, patients with significant medical comorbidities present a further challenge given the risks of a major surgical procedure. Simple skin graft directly onto bone frequently fails and does not provide durable coverage, while combined artificial skin substitute and split-thickness skin graft approach involves a prolonged treatment period with multiple-staged procedures. Herein, we present a case of an 82-year-old female patient with complex medical comorbidities with a large scalp defect and calvarial exposure following Moh's surgery. She was successfully treated with a single-stage acellular dermal matrix and a STSG reconstruction. Her treatment period was effectively shortened, and she had an excellent clinical outcome.

## CASE PRESENTATION

**Figure 1:**
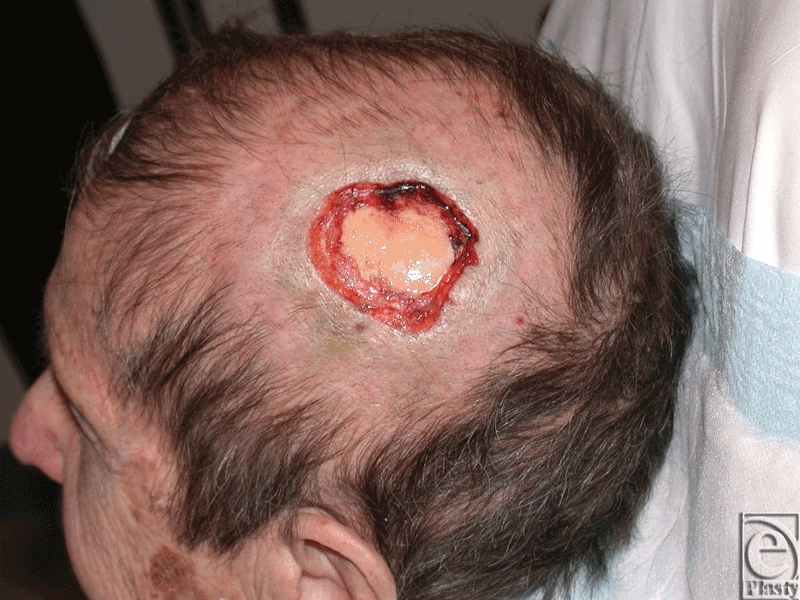
Full-thickness scalp wound with exposed calvarium following Moh's resection.

An 82-year-old woman presented with a full-thickness scalp defect following Moh's resection. She had a complex medical history including metastatic thymoma to lungs and spine treated with multiple surgical resections, chemotherapy and radiation, chronic obstructive pulmonary disease requiring oxygen therapy, aortic stenosis, congestive heart failure, and hypertension. In April 2010, she underwent Moh's resection of an infiltrating BCC from the left parietal scalp. The resection included the periosteum layer resulting in a 5-cm scalp wound with a 3.5-cm wide exposed calvarium (Fig 1). The surrounding scalp demonstrated minimal laxity, and the patient was reported to be a poor candidate for anesthesia or prolonged surgical procedures due to her comorbidities. She was initially treated with a vacuum-assisted closure (VAC) dressing but did not tolerate this approach secondary to pain associated with VAC dressing change. The decision was made to proceed with definitive surgical reconstruction. Although reconstruction using artificial skin substitute (Integra; Life Sciences, Plainsboro, New Jersey) was initially considered, the prolonged period of local wound care required and the need for a second-stage STSG made this approach less appealing. Thus, a simpler single-stage approach using acellular dermal matrix (ADM) and STSG was planned.

**Figure 2:**
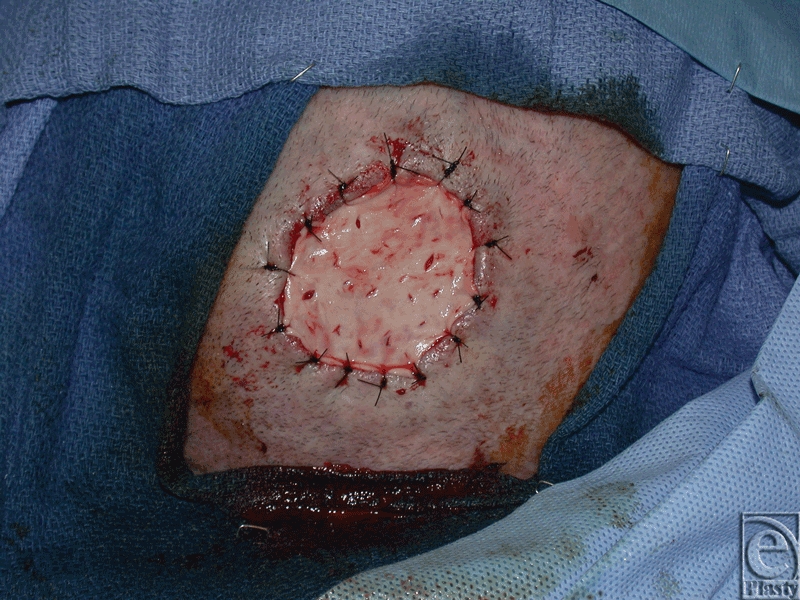
Acellular dermal matrix inset intraoperatively after burring of the outer table.

**Figure 3:**
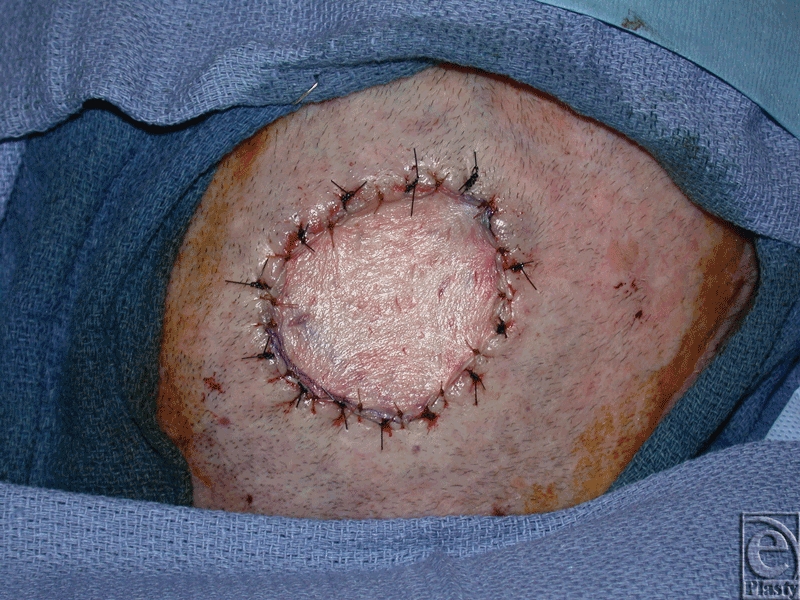
Split-thickness skin graft inset intraoperatively onto the acellular dermal matrix.

Under general anesthesia, the scalp wound edges were sharply debrided, and the outer table of calvarial bone was burred down to a bleeding base exposing the vascular diploic space. A thin sheet (0.53–0.76 mm) of ADM (AlloDerm; Lifecell, Branchburg, New Jersey) was reconstituted and inset into the wound base using 3-0 nylon sutures (Fig 2). A 0.011-inch STSG was then harvested from the thigh and inset on top of the ADM with 4-0 chromic sutures. Both the ADM and STSG were piecrusted to prevent hematoma and seroma (Fig 3). A nonadherent dressing and VAC dressing set at 100 mm Hg continuous therapy were applied.

**Figure 4:**
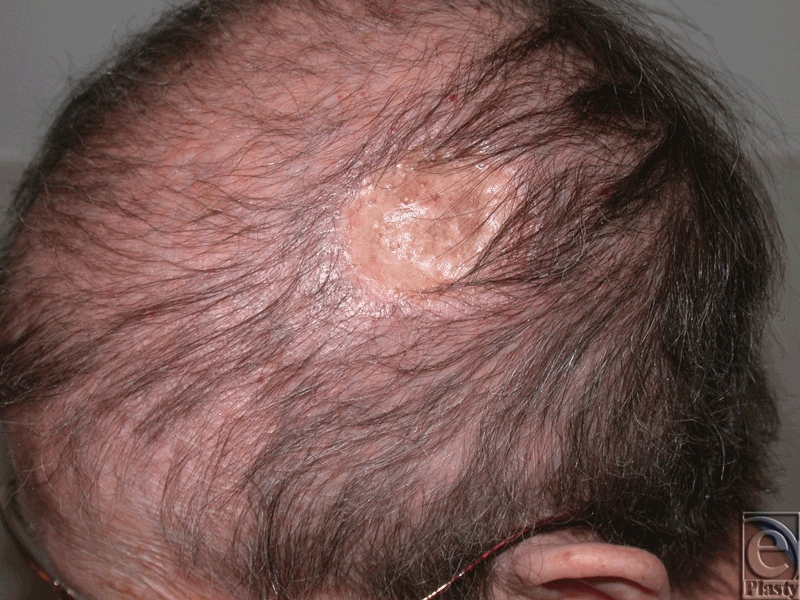
Three-months postoperatively, with 100% take and stable coverage.

The VAC was removed on postoperative day 7, and the ADM and STSG were noted to be stably intact with evidence of revascularization and without hematoma, seroma, or infection. A second cycle of VAC was applied for 3 additional days, and then the skin graft was dressed with a routine Xeroform (Kendall/Covidien, Mansfield, MA) and dry sterile dressing. At 3 months' follow-up, the patient demonstrated 100% graft take and achieved successful scalp reconstruction (Fig 4).

## DISCUSSION

A broad armamentarium of reconstructive options exists for the treatment of scalp defects including skin grafts, tissue expansion, local flaps, or free tissue transfer.^1^,^2^ Because of the thin subcutaneous tissue of the scalp, safety considerations often lead Moh's surgeons to include the pericranium as part of wide local excisions for cutaneous malignancies of the head.^3^ The resultant full-thickness scalp defects with underlying exposed calvarium pose a particular reconstructive challenge.

Durable soft tissue coverage remains essential in full-thickness scalp defects to prevent fluid and protein loss, calvarial desiccation, and infection. The exposed skull bone, with its poor vascularity, is slow to form granulation tissue, and wounds are unlikely to achieve satisfactory healing by secondary intention. Moreover, resulting wound contraction from secondary healing may yield unstable coverage and undesirable cosmetic outcome. Local or free flaps are generally considered to be the best reconstructive options; however, they have inherent surgical risks, particularly in elderly patients with systemic comorbidities.^4^ The relative inelasticity of the scalp requires wide undermining and creation of large rotational flaps even for relatively small defects.^1^ Skin graft placed directly onto debrided bone, although simpler in technique, does not provide durable coverage and frequently leads to poor graft take with recurrent ulcerations.^5^

Recent innovation in the treatment of full-thickness scalp defects has been the use of artificial dermis. There are a number of published reports describing this application with the majority focusing on the use of Integra.^6^^-^7,9 Integra is a bilaminate synthetic construct consisting of an outer silicone layer as an epidermal substitute and an inner collagen-glycosaminoglycan, which serves as a dermal regeneration template. A major disadvantage, however, involves the need for a second-stage procedure along with a prolonged period of local wound management for several weeks prior to skin grafting.^10^ Complications such as seroma and infection can occur during the wound management period resulting in treatment delays or eventual failure. Most recently, a 1-stage facial reconstruction using a newly developed single layer dermal regeneration template has been reported.^11^ While this may eventually emerge as a promising treatment option, current clinical data is limited. An alternative artificial dermis is ADM derived from human cadaver skin, which can provide a dermal scaffold for vascular invasion. Although use of ADM for soft tissue defects also generally requires a staged approach where ADM reconstruction is followed by a second-stage STSG, Jung et al^12^ reported the first case of a single-stage split-thickness grafting of exposed calvarium with ADM.

Our case supports this treatment option by illustrating another successful case in which a complex scalp defect was quickly and effectively managed using a single-stage ADM and STSG. This simple technique can be particularly useful in patients with medical comorbidities that make more extensive surgical treatment risky. Flap reconstructions can result in considerable morbidity, such as flap necrosis, donor-site morbidity, scarring, and prolonged surgical procedure and recovery. The use of ADM and STSG in a single stage can eliminate the need for large regional or free flaps while achieving complete coverage in a much simpler fashion and with minimal morbidity.

## SUMMARY

Full-thickness scalp defects with exposed calvarium pose a reconstructive challenge, particularly in patients with extensive comorbidities. A single-stage ADM and STSG reconstruction represents a simple and valuable surgical approach to achieving durable scalp coverage without requiring a donor-site or prolonged treatment.
